# Support Pressure Acting on the Epicardial Surface of a Rat Left Ventricle—A Computational Study

**DOI:** 10.3389/fcvm.2022.850274

**Published:** 2022-07-06

**Authors:** Denisa Martonová, David Holz, Dorothea Brackenhammer, Michael Weyand, Sigrid Leyendecker, Muhannad Alkassar

**Affiliations:** ^1^Institute of Applied Dynamics, Friedrich-Alexander-Universität Erlangen-Nürnberg, Erlangen, Germany; ^2^Department of Cardiac Surgery, Friedrich-Alexander-Universität Erlangen-Nürnberg, Erlangen, Germany

**Keywords:** cardiac assist device, cardiomyopathy, epicardial heart support, left ventricle, support pressure, fibrosis

## Abstract

The present computational study investigates the effects of an epicardial support pressure mimicking a heart support system without direct blood contact. We chose restrictive cardiomyopathy as a model for a diseased heart. By changing one parameter representing the amount of fibrosis, this model allows us to investigate the impairment in a diseased left ventricle, both during diastole and systole. The aim of the study is to determine the temporal course and value of the support pressure that leads to a normalization of the cardiac parameters in diseased hearts. These are quantified *via* the end-diastolic pressure, end-diastolic volume, end-systolic volume, and ejection fraction. First, the amount of fibrosis is increased to model diseased hearts at different stages. Second, we determine the difference in the left ventricular pressure between a healthy and diseased heart during a cardiac cycle and apply for the epicardial support as the respective pressure difference. Third, an epicardial support pressure is applied in form of a piecewise constant step function. The support is provided only during diastole, only during systole, or during both phases. Finally, the support pressure is adjusted to reach the corresponding parameters in a healthy rat. Parameter normalization is not possible to achieve with solely diastolic or solely systolic support; for the modeled case with 50% fibrosis, the ejection fraction can be increased by 5% with purely diastolic support and 14% with purely systolic support. However, the ejection fraction reaches the value of the modeled healthy left ventricle (65.6%) using a combination of diastolic and systolic support. The end-diastolic pressure of 13.5 mmHg cannot be decreased with purely systolic support. However, the end-diastolic pressure reaches the value of the modeled healthy left ventricle (7.5 mmHg) with diastolic support as well as with the combination of the diastolic and systolic support. The resulting negative diastolic support pressure is −4.5 mmHg, and the positive systolic support pressure is 90 mmHg. We, thereby, conclude that ventricular support during both diastole and systole is beneficial for normalizing the left ventricular ejection fraction and the end-diastolic pressure, and thus it is a potentially interesting therapy for cardiac insufficiency.

## 1. Introduction

Cardiovascular diseases remain the leading cause of death worldwide (1). If conservative therapy is inadequate due to the pathological condition of the heart, there are usually only two options remaining to treat this condition: cardiac assist devices or heart transplantation. However, there is a chronic shortage of donors. For example, in Germany, 320 patients received a donor heart in 2020, while the remaining 700 patients were still on the waiting list at the end of that year (2). While heart transplantation is a superior option in terms of survival and functional capacity, significant improvements in the field of cardiac assist devices have resulted in promising solutions that close the gap between availability and demand for donor's hearts. Over the last decades, various types of mechanical pumping systems, known as left ventricular (LV) assist devices, have been developed (3). They are used as an invasive form of therapy to directly support blood circulation. In principle, these systems mostly work to bypass the weakened heart. Such cardiac assist devices can relieve the load on the heart and become either a short- to medium-term bridge to treat cardiac insufficiency until possible transplantation (bridge-to-transplantation) or a permanent solution (destination therapy). Despite the rapid development of new devices and improvements in post-surgery survival and functional capacity nearing those of heart transplantation, there are still many problems to solve, including bleeding, thrombosis, strokes, and infections (3, 4).

For the most part, these problems are caused by the direct contact of blood with the cardiac assist device. Several approaches to avoiding direct blood contact, commonly referred to as direct cardiac compression devices, have been proposed to overcome such difficulties. The first bridge-to-transplantation based on a pneumatic compression cup (5) was followed by several other innovative approaches, e.g., (6–9). Review articles provide a more detailed overview of available direct cardiac compression devices (3, 10). For example, systems from AdjuCor GmbH (8) and CorInnova Inc. (9), currently in the preclinical testing phase, provide support during systole and are minimally invasive implants. The common principle of these devices is that they develop a pressure (force per area) that acts on the epicardial surface. Theoretically, a diseased heart can be thereby supported solely in the diastole, solely in the systole, or in both cardiac phases. However, most direct cardiac compression devices only work during systole. The objective of our computational study is to investigate (1) if an application of support pressure on the LV during both the diastolic and systolic phases is able to normalize the ejection fraction (EF) and left ventricular end-diastolic pressure (EDP) and (2) how much pressure is needed. The current study does not focus on the modeling of a particular cardiac assist device.

In general, the functionality of a direct cardiac compression device is mainly determined by the improvement in the pump function of a diseased heart. In the present study, it is quantified *via* the EF and EDP. However, the influence of a direct cardiac compression device on cardiac performance is not straightforward to compute, due to, e.g., the varying mechanical properties of active biological cardiac tissue that is undergoing complex deformations. The complex orthotropic tissue structure of the healthy myocardium, which can be modeled with different approaches (11–13), plays an important role. Furthermore, the amount of fibrosis in the ventricular wall strongly influences cardiac performance. Over the course of many heart diseases, there is a remodeling process that leads to an increase in fibrosis (14, 15). This is often independent of the triggering disease and can occur after cardiac volumetric pressure loading or ischemia (16). Various studies indicate a correlation between diastolic function and the amount of myocardial fibrosis (17, 18). Regardless of the functional impact of primary cardiomyopathy, the fibrosis progressively limits the diastolic function of the ventricle (19). As long as the condition can be systolically compensated, there is no reduction in the overall myocardial function (heart failure with preserved ejection fraction) (20). However, if the amount of fibrosis exceeds a certain level, the reduction in the diastolic function can no longer be systolically compensated, and the overall myocardial function is reduced (19). It has been shown that ventricular fibrosis inversely correlates with the ejection fraction, both in rats (21) and humans (17). Since the functional impairment resulting from the diastolic dysfunction due to myocardial fibrosis is the terminal stage of most cardiac diseases (17), a better understanding of the role of diastolic function and its relationship with the amount of fibrosis is important. We, therefore, calculated the effects of increasing fibrosis on cardiac function in a computational model of restrictive cardiomyopathy. Studies using postmortem mechanical testing in animal models after myocardial infarction have shown that the fibrosis leads to the stiffening of the cardiac tissue (22–25).

To optimally support a diseased heart *via* a direct cardiac compression device, two main factors play an important role: the time evolution of the force generated by the device and its maximum and minimum values. To date, how these factors influence cardiac function has not been investigated in detail. In this early research study, a computer simulation offers many advantages. First, it saves time compared to experimental testing. Second, there are beneficial synergies between computational modeling and simulation and experimental testing; for example, various parameters can be predicted that cannot be directly measured. Third, modeling and simulation can eventually be used to improve the adaptivity of such a system at a patient-specific level, making it a fundamental instrument in modern and future medicine. So far, a few finite element-based computational models that account for the coupling between a cardiac assist device and the heart have been developed. The existing work presents simulations for the ventricular pumps that are coupled with a univentricular (26) or biventricular heart models (27) *via* a cannula. A computational model for the innovative support system from AdjuCor GmbH is presented in Hirschvogel et al. (28). Recently, Chavanne (29) presented a simulation of a dielectric elastomer actuator-based aortic plaster interacting with a lumped parameter model for the heart.

The present study uses a finite element-based computer simulation. The study models an actively contracting rat LV with different amounts of fibrosis (30) and investigates the influence of direct cardiac compression devices supporting the LV on cardiac performance during both diastolic and systolic phases. As the current study does not focus on modeling a particular cardiac assist device, for simplicity, the model represents a support pressure acting on the outer surface of the modeled LV, as depicted in Figure 2A. During diastole, a negative support pressure is modeled to facilitate the ventricular filling, whereas a positive support pressure in systole is applied to eventually support the blood outflow from the LV. In particular, we investigate whether a support pressure calculated as a difference between the modeled ventricular pressure in a fibrotic and healthy LV would lead to a normalization of cardiac function parameters. Subsequently, the maximum positive systolic and negative diastolic support pressures are optimized such that the EF, EDP, and left ventricular end-diastolic volume (EDV) of a control healthy rat LV are restored. In the present study, a simplified rat LV ventricle is computationally modeled.

## 2. Methods

This section describes the computational model of the contraction of a rat LV and the associated numerical experiments.

### 2.1. Modeling

#### 2.1.1. Balance Equations and Support Pressure Boundary Conditions

The field equation governing the state of the material point ***X***∈Ω_0_ at time *t*, *t*∈[*t*_0_, *t*_*f*_] (*t*_0_ and *t*_*f*_ are the initial and final times, respectively) can be formulated. The mechanical field equation is the balance of linear momentum together with the boundary conditions on the boundaries Γ_φ_, Γ_1_, and Γ_2_:


(1) $$0=\text{Div}[F\;\cdot \;S]+F^{\varphi }\text{ in }\Omega_{0},$$

(2) $$\varphi (X,t)=\bar{\varphi }\text{ in }\Gamma_{\varphi },\;T(X,t)=-p_{i}JF^{-T}N\text{ in }\Gamma_{i},i\in \{1,2\}$$

where **F** is the deformation gradient with its determinant *J* = det**F**, **S** is the second Piola Kirchhoff stress (PK2), ***F***^φ^ is the external mechanical body force, ***φ*** is the displacement with prescribed value $\bar{\varphi }$ on the boundary Γ_φ_, ***T*** is the surface traction vector in the reference configuration, ***N*** is the outer unit normal in the reference configuration and *p*_*i*_ are the prescribed values of the pressures acting on the boundaries Γ_*i*_, *i*∈{1, 2}. The pressure *p*_1_ in the LV, obtained from the three-element Windkessel model (representing the interaction between the LV, aorta, and peripheral arteries), serves as a Neumann boundary condition on the endocardial surface Γ_1_ whereas the support pressure *p*_2_ serves as a Neumann boundary condition on the epicardial surface Γ_2_. The basis of the LV (Γ_φ_) is fixed in the longitudinal direction; additionally, the nodes on the outer basis are fixed in all directions (31).

#### 2.1.2. Constitutive Equations

In the present study, the total PK2 is additively decomposed into the passive part **S**_*pas*_ and the active part **S**_*act*_ (31–35), namely


(3) $$\begin{array}{r} \text{S}={\text{S}}_{pas}+{\text{S}}_{act}. \end{array}$$

Based on Martonová et al. (23), we model the LV as a mixture of the intact myocardium and fibrotic scar structure. The amount of fibrosis, *fib*, serves as a scaling factor. Furthermore, we assume that only the intact muscle tissue is able to contract (30, 36), and therefore the active part of the stress tensor is as well-scaled by the amount of fibrosis. The resulting PK2 reads as


(4) $$\begin{array}{l} S=fib\;S_{pas}^{s}+(1-fib)(\;S_{pas}^{m}+S_{act}^{m}), \end{array}$$

where the superscripts *s* and *m* correspond to the scar and intact myocardium and the subscripts *pas* and *act* to the passive and active parts of the PK2, respectively. In particular, by setting *fib* = 0, only the intact cardiac tissue is modeled. As proposed in Martonová et al. (23) for the passive part, the scar structure is modeled as a transversely isotropic material and the intact myocardium as an orthotropic material, according to Holzapfel and Ogden (37). The active contraction of the intact myocardium is modeled following the simple time-dependent approach from Pfaller et al. (38):


(5) $$\begin{array}{r} {\text{S}}^{act}\left(t,f_{0},n_{0}\right)=T\left(t\right)\left(f_{0}⊗f_{0}+\nu n_{0}⊗n_{0}\right). \end{array}$$

**Figure 1:**
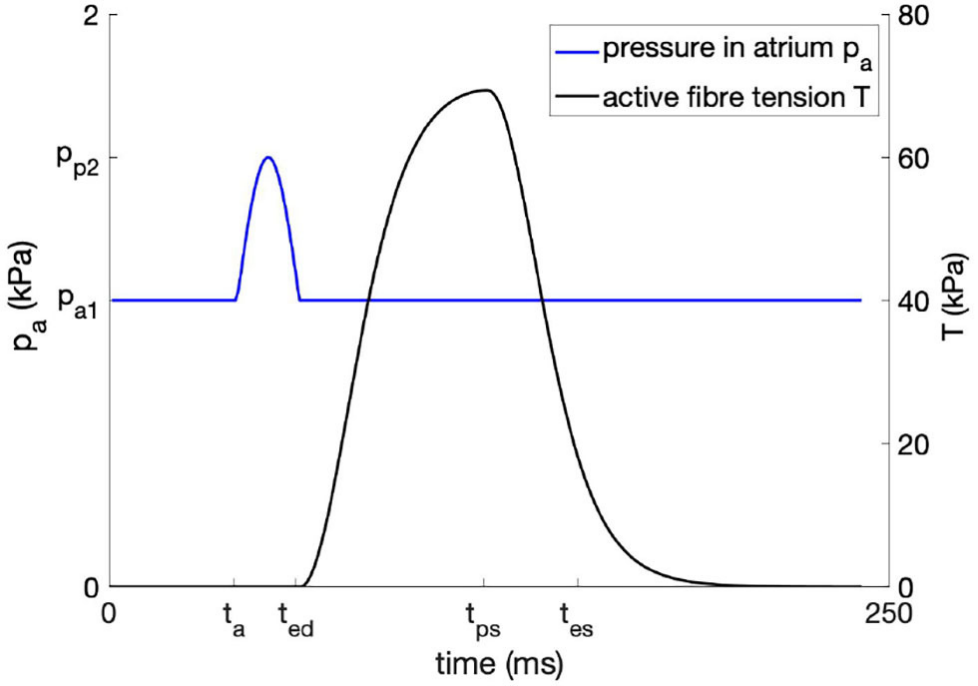
Temporal evolution of the time-dependent atrial pressure and the active fiber tension.

The temporal evolution of the active tension *T* depicted in Figure 1 is obtained by using the parameters shown in Table A1. For the equations describing *T*(*t*), we refer to the Appendix or the original study (38).

Based on the experimental evidence (39) and our previous study (30), in addition to the contraction in the fiber direction ***f***_0_, reduced active stress along the cross-fiber direction ***n***_0_ is added and scaled by ν in Equation (5). We note that the electromechanical coupling is omitted in this study as it would introduce further complexity and variability. However, the model can be coupled with a model for electrical excitation (38). The constitutive model is applied to the generic ellipsoidal rat LV based on the data from echocardiography. For more details regarding the geometry, fiber orientation, compressibility, and the Windkessel model serving as a boundary condition in Equation (1), we refer to previous study (35).

#### 2.1.3. Diastolic Filling

The blood flow *I* between the left atrium and LV is given as


(6) $$\begin{array}{r} I=\frac{\Delta p}{R_{v1}}, \end{array}$$

where Δ*p* is the pressure difference between the left atrium and LV, and *R*_*v*1_ is the resistance of the atrioventricular valve. The pressure in the atrium *p*_*a*_ is modeled according to the following equation:


(7) $$\begin{array}{l} p_{a}(t)=\{\begin{array}{ll} (1+fib)p_{a1}\; & \text{if }t\leq t_{a}\\ (1+fib)(p_{a1}+p_{a2}\sin(t-t_{a}))\; & \text{if }t_{a}<t\leq t_{ed}\\ (1+fib)p_{a1}\; & \text{if }t>t_{ed} \end{array} \end{array}$$

where *p*_*a*1_ and *p*_*a*2_ are the minimum and maximum atrial pressures, and *t*_*a*_, *t*_*ed*_ model the onset of the atrial and ventricular contraction, respectively. The resulting curve is shown in Figure 1. We note that the atrial pressure is as well-scaled by the amount of fibrosis, allowing us to model the higher EDP observed in rats with different amounts of fibrosis after myocardial infarction (40). To avoid an unlimited blood inflow from the atrium to the LV, the maximal EDV is restricted to that of the control rat.

### 2.2. Numerical Experiments

In the following, *p*(*t*) defines the support pressure acting on the epicardial surface of the LV. Three numerical experiments are performed.

First, the amount of fibrosis is varied from 0% to 60% in order to compare the cardiac performance represented by the EF in the fibrotic rat LV at different fibrosis stages without a support pressure.

**Figure 2:**
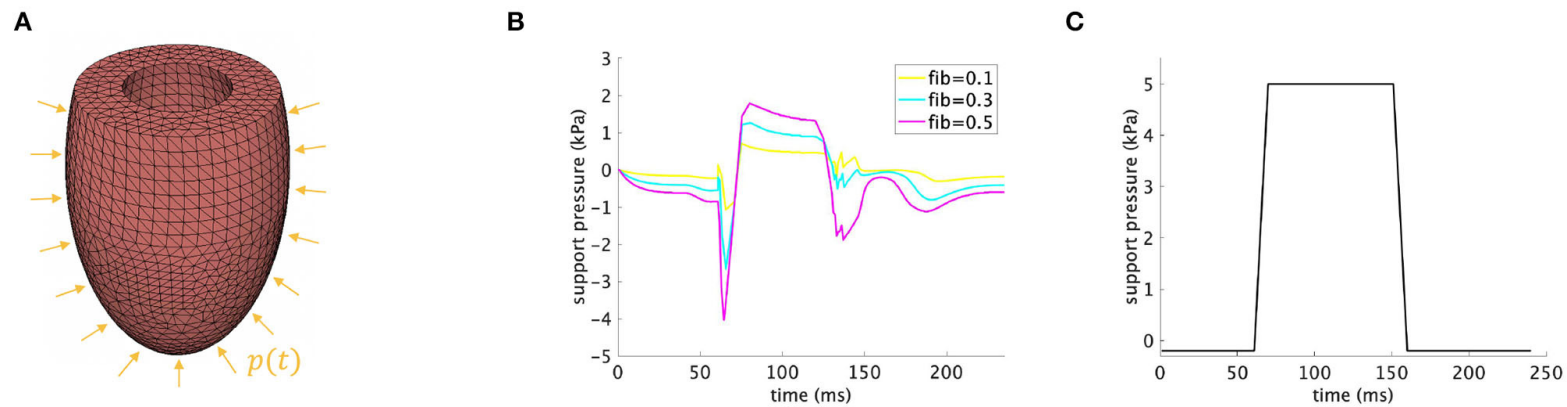
**(A)** Discretized model of the rat LV with applied support pressure *p*(*t*) on the epicardial surface. **(B)** Time-dependent support pressure according to Equation (8) for *fib*∈{0.1, 0.3, 0.5}. **(C)** Time-dependent support pressure according to Equation (9).


Second, a support pressure is applied in order to increase the diastolic and systolic performance of the diseased LV at different fibrosis stages. The support pressure is computed as a difference between the left ventricular pressure in the control and diseased LV at each time point during the cardiac cycle (Figure 2B), namely

(8) $$\begin{array}{r} p\left(t\right)=p_{LV}^{0}\left(t\right)-p_{LV}^{fib}\left(t\right), \end{array}$$

where $p_{LV}^{0}$ is the pressure in the control LV (*fib* = 0), and $p_{LV}^{fib}$ is the left ventricular pressure in a fibrotic LV for different amounts of fibrosis. In these examples, we consider *fib*∈{0.1, 0.3, 0.5}.

Third, the support pressure displayed in Figure 2C and obeying the following equation is applied on exemplary fibrotic LVs, namely *fib*∈{0.3, 0.5}.


(9) $$\begin{array}{l} p(t)=\{\begin{array}{ll} p_{min}\; & \text{if }t\leq t_{ed}\\ p_{min}+\frac{p_{max}-p_{min}}{t_{\Delta }}(t-t_{ed}) & \text{if }t_{ed}<t\leq t_{ed}+t_{\Delta }\\ p_{max}\; & \text{if }t_{ed}+t_{\Delta }\leq t\leq t_{es}\\ p_{max}-\frac{p_{max}-p_{min}}{t_{\Delta }}(t-t_{es}) & \text{if }t_{es}<t\leq t_{es}+t_{\Delta }\\ p_{min}\; & \text{if }t>t_{es}+t_{\Delta } \end{array}\% \end{array}$$

As mentioned in the Introduction, three possibilities for supporting a weakened heart can be distinguished: pure diastolic, pure systolic, and combined diastolic and systolic support. For the first possibility, we aim to regain EDP^*^ and EDV^*^ of the healthy control LV (within given tolerances *tol*_1_, *tol*_2_). For the second and third possibilities, we additionally aim to nearly reach ESV^*^ of the healthy control LV. Note that EDP and EDV depend on the value of *p*_*min*_ only, i.e., *EDP*(*p*_*min*_), *EDV*(*p*_*min*_), while ESV depends on both values *p*_*min*_ and *p*_*max*_, i.e., *ESV*(*p*_*min*_, *p*_*max*_). We started with the determination of the optimal negative support pressure during the diastole $p_{min}=p_{min}^{*}$ in Equation (9). In a second step, only systolic support is considered, i.e., *p*_*min*_ in Equation (9) is set to zero, and the maximal positive support pressure $p_{max}=p_{max-sys}^{*}$ is determined. In the last step, a combination of diastolic and systolic support is assumed. Therefore, $p_{min}^{*}$ from the first step is fixed, and the optimal systolic support $p_{max}=p_{max}^{*}$ is to be found. We note that $p_{max-sys}^{*}\neq p_{max}^{*}$ hold in general, as the end-diastolic states are different in both cases. For these three steps, the following algorithm is performed:

1. Diastolic support: Find $p_{min}^{*}$ so that $|EDV\left(p_{min}^{*}\right)-EDV^{*}|\leq tol_{1}$ and $|EDP\left(p_{min}^{*}\right)-EDP^{*}|\leq tol_{2}$ are fulfilled
2. Systolic support: Set *p*_*min*_ = 0 kPa and find $p_{max-sys}^{*}$ so that $|ESV\left(0\text{kPa},p_{max-sys}^{*}\right)-ESV^{*}|\leq tol_{1}$ is fulfilled
3. Diastolic and systolic support: Set $p_{min}=p_{min}^{*}$ and find $p_{max}^{*}$ so that $|ESV\left(p_{min}^{*},p_{max}^{*}\right)-ESV^{*}|\leq tol_{1}$ is fulfilled

where the optimal support pressure p^*^(*t*) from Equation (9) is determined via the negative diastolic support pressure $p_{min}^{*}$ and positive systolic support pressures $p_{max-sys}^{*},p_{max}^{*}$, for the purely systolic support and systolic support combined with diastolic support, respectively.

## 3. Results

Different quantities characterizing the cardiac performance are plotted for all simulations introduced in Section 2.2, including pressure volume loop, EF, EDV, ESV, EDP, and averaged end-diastolic hydrostatic stress over the domain, that is $\sigma_{H}=\frac{1}{n_{el}}\overset{n_{el}}{\underset{i=1}{\sum }}\overset{3}{\underset{j=1}{\sum }}\frac{\sigma_{jj}^{i}}{3}$, where $\sigma_{jj}^{i}\left(j=1,2,3\right)$ are the diagonal components of the Cauchy stress in the *i*-th finite element and *n*_*el*_ = 22846 is the total number of the tetrahedral finite elements in the computational domain representing the LV; refer to Figure 2A. We note that EDP is the fluid pressure inside the cavity of the modeled LV, whereas the end-diastolic hydrostatic stress, computed according to the above formula, depends on the myocardial tissue structure and its volume change during the deformation (41). The latter can be interpreted as a measure of the force that drives fluid out of the myocardium and into the surrounding tissues. Positive hydrostatic stress means that the tissue is under extension and there is an increase in its volume (fluid flows into the myocardium), whereas negative hydrostatic stress implies that the myocardial tissue is compressed (fluid flows out of the myocardium).

### 3.1. Different Amounts of Fibrosis Without Support

**Figure 3:**
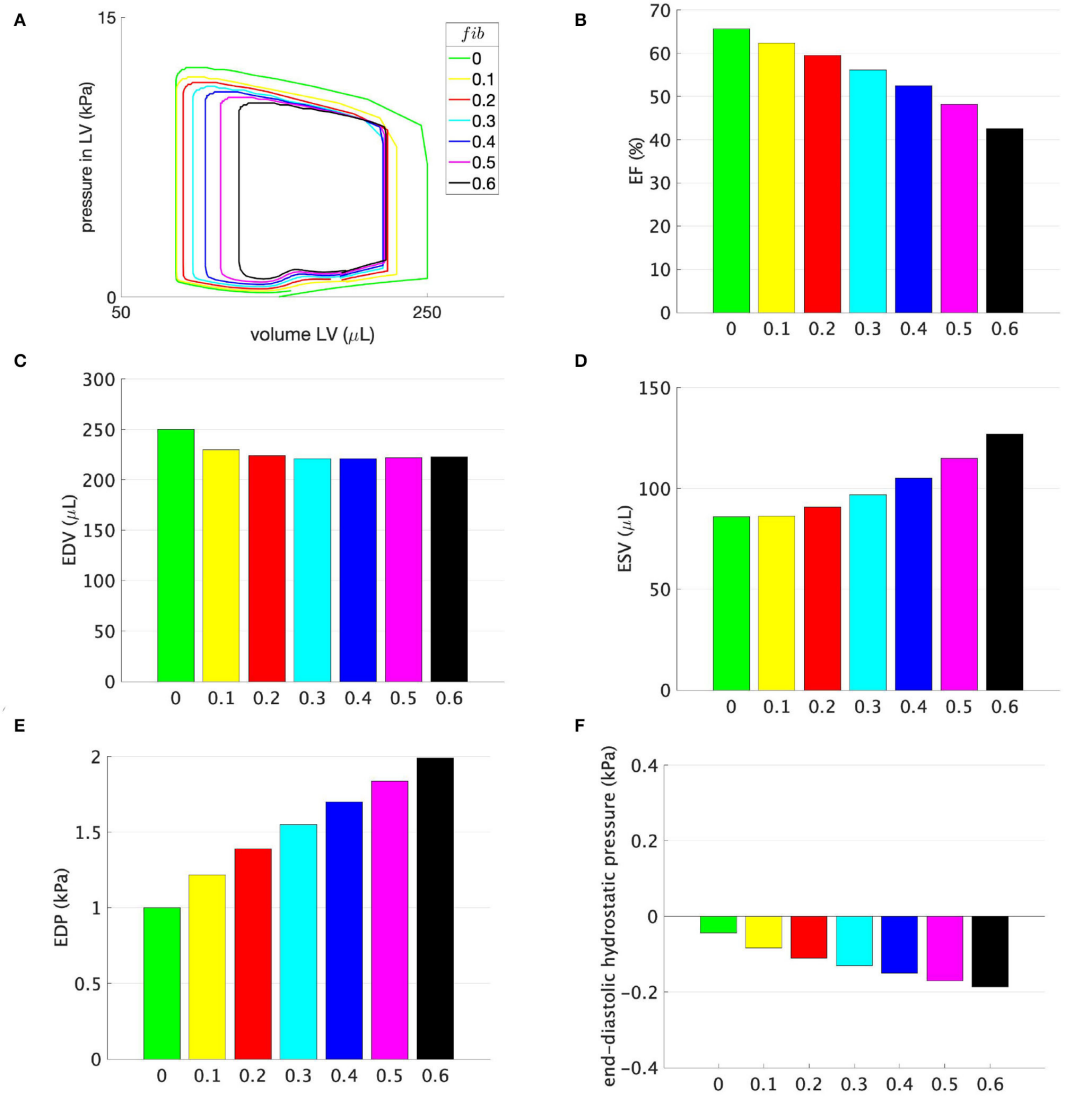
**(A)** Pressure-volume loops, **(B)** ejection fraction (EF), **(C)** EDV, **(D)** ESV, **(E)** EDP, and **(F)** end-diastolic hydrostatic stress for models with different amounts of fibrosis *fib* displayed on the horizontal axis. No support pressure is applied.

Figure 3 shows the simulation results for scenarios with different amounts of fibrosis without any support pressure. Clearly, by increasing the amount of fibrosis, EF and EDV decrease, whereas ESV and EDP increase. For example, EF and EDP in the healthy model are 65.6% and 1 kPa (7.5 mmHg), respectively. These are close to the experimentally reported values in rats, which are slightly above the normal values in humans (35, 42, 43). By increasing the amount of fibrosis to 30% and 50%, EF reduces to 56.1% and 46.1%, whereas EDP increases to 1.6 kPa (12 mmHg) and 1.8 kPa (13.5 mmHg), respectively; refer to Figures 3B,E.

### 3.2. Support Pressure as the Difference With Respect to the Control Rat (*fib* = 0)

**Figure 4:**
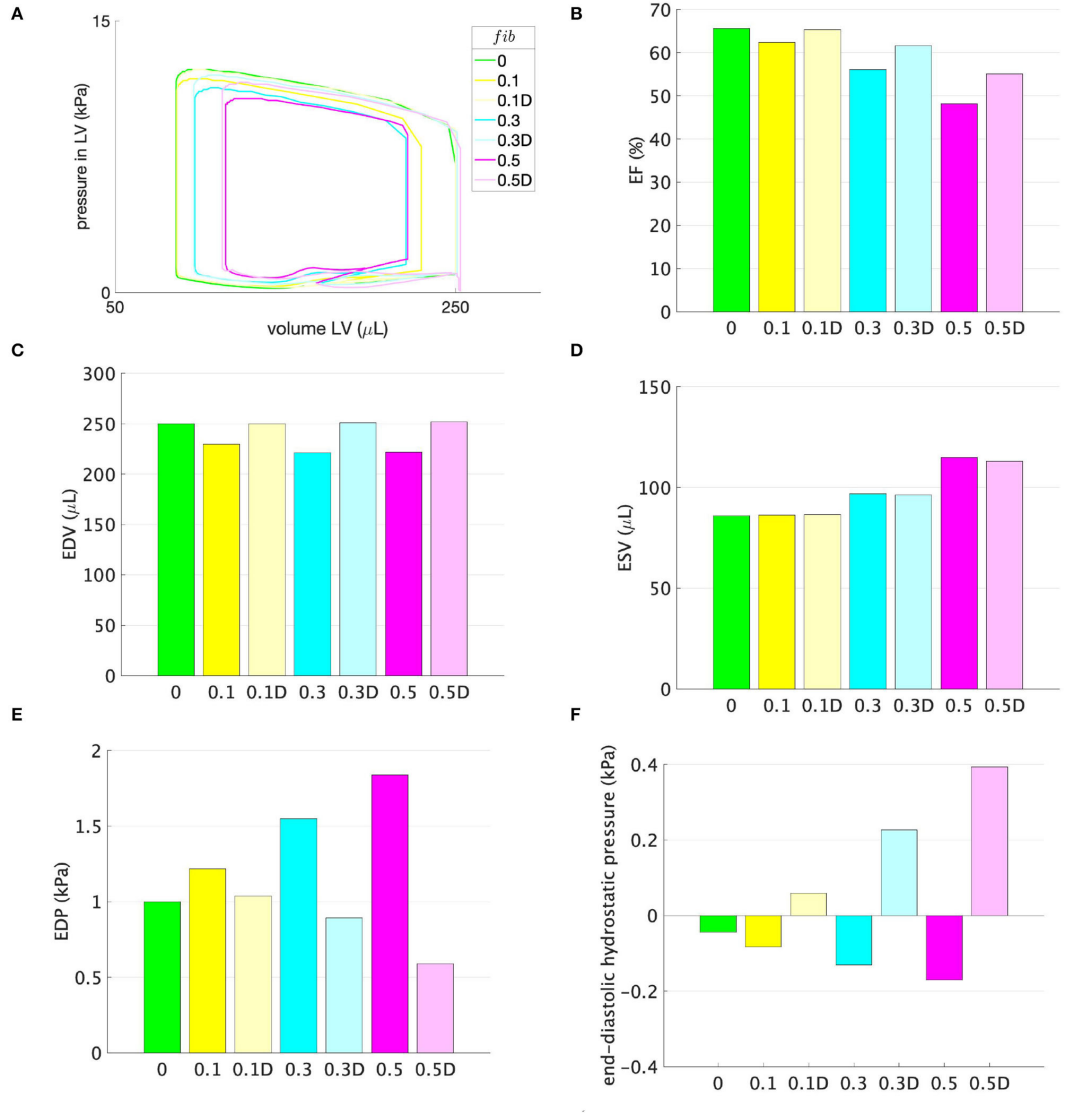
**(A)** Pressure-volume loops, **(B)** EF, **(C)** EDV, **(D)** ESV, **(E)** EDP, and **(F)** end-diastolic hydrostatic stress for models with different amounts of fibrosis *fib* displayed on the horizontal axis. No support pressure is applied for models marked with the corresponding amount of fibrosis *fib* and displayed in opaque colors, whereas support pressure according to the function shown in Figure 2B, is applied for models with the suffix D displayed in transparent colors.

In Figure 4, changes in the cardiac function parameters are displayed for the rats with 10%, 30%, and 50% fibrosis in the LV. By applying a support pressure, computed according to Equation (8) and displayed in Figure 2B, EF, EDV, EDP, and end-diastolic hydrostatic pressure are at least partially improved, refer to Figures 4B,C,E,F, respectively. ESV remains nearly unchanged, refer to Figure 4D.

### 3.3. Constant Minimal and Maximal Support Pressure

**Figure 5:**
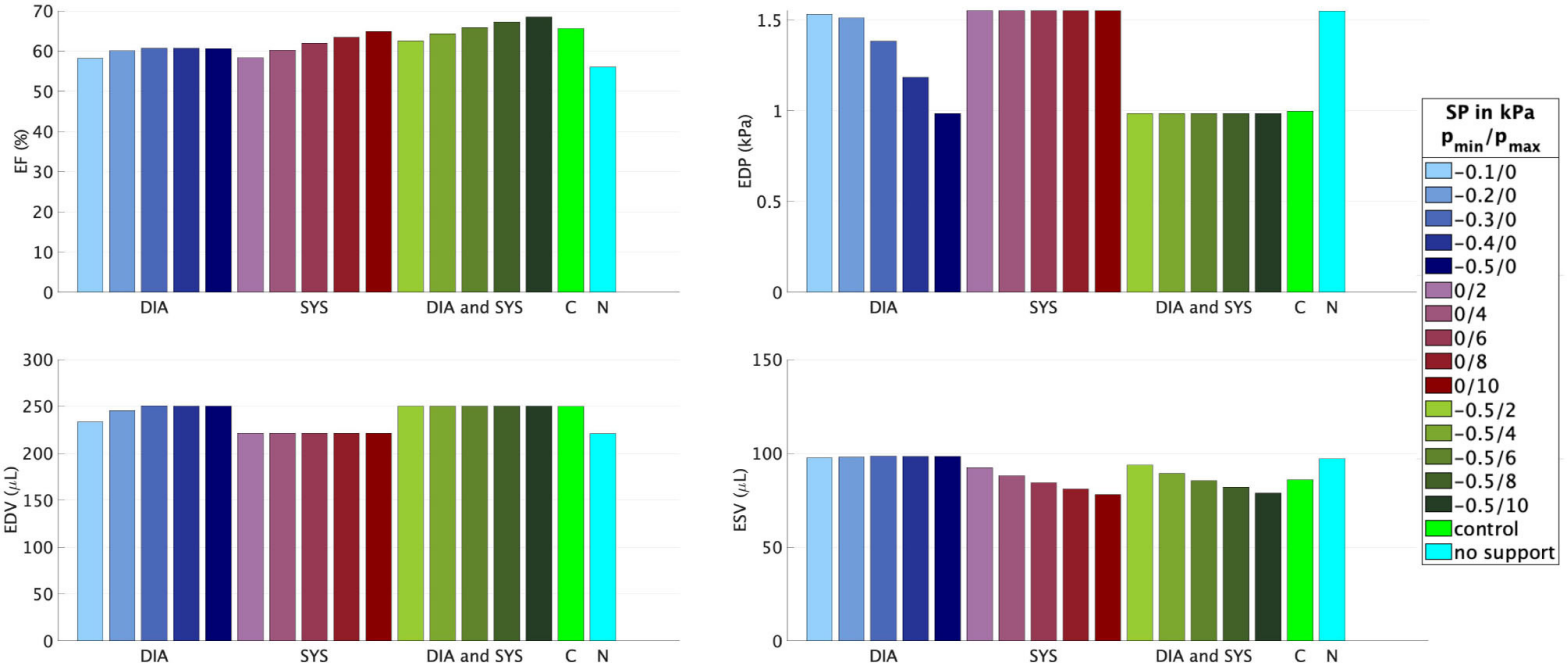
Ejection fraction, EDP, EDV, and ESV for different combinations of the minimal *p*_*min*_ and maximal *p*_*max*_ values of the support pressure (SP) applied to the epicardial surface of the model with *fib* = 0.3. DIA, diastolic support (blue); SYS, systolic support (red); DIA and SYS, combined support (green); C, control rat LV (light green); N, rat LV with *fib* = 0.3 without any support (blue-green).

**Figure 6:**
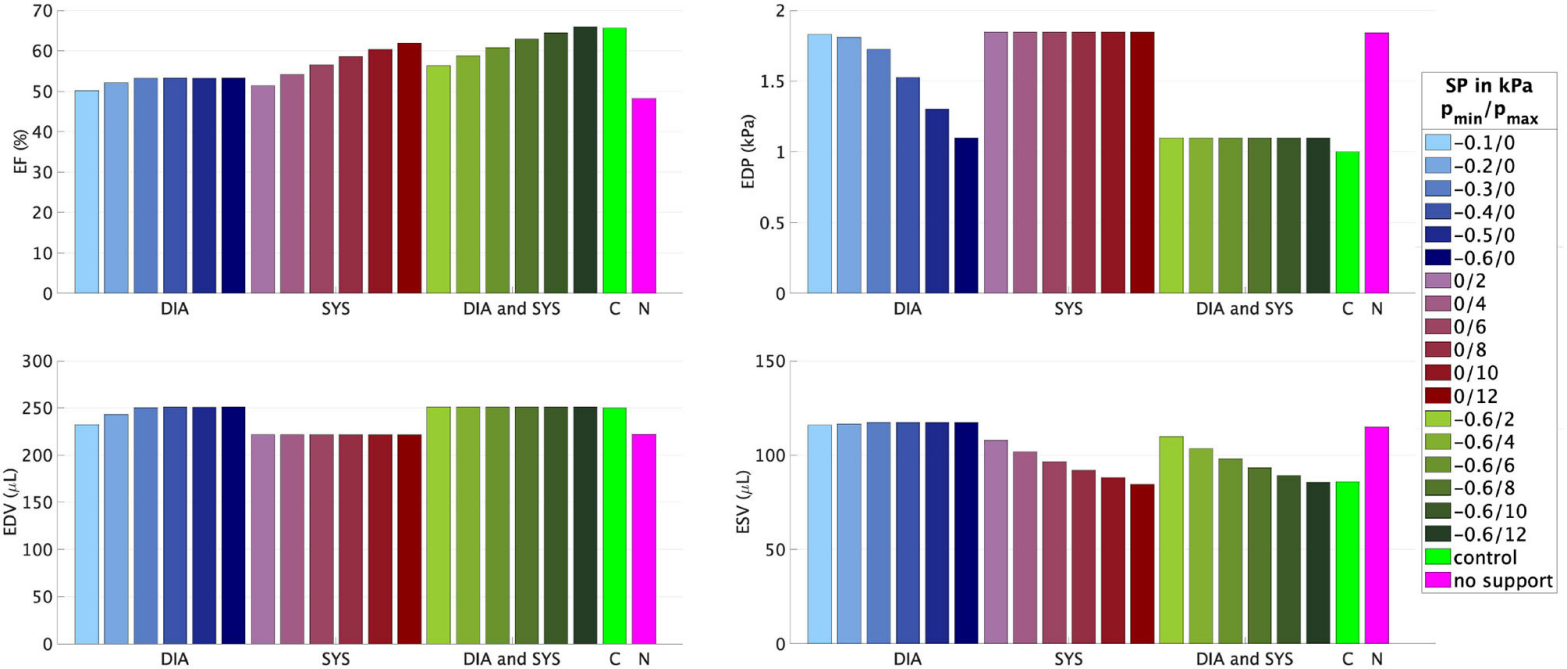
Ejection fraction, EDP, EDV, and ESV for different combinations of the minimal *p*_*min*_ and maximal *p*_*max*_ values of the support pressure (SP) applied to the epicardial surface of the model with *fib* = 0.5. DIA, diastolic support (blue); SYS, systolic support (red); DIA and SYS, combined support (green); C, control rat LV (light green); N, rat LV with *fib* = 0.5 without any support (pink).

Figures 5, 6 illustrate how the stepwise increase in the negative diastolic and positive systolic support pressure influences cardiac performance. The support pressure is increased until the EDP, EDV, and ESV of the control rat are reached up to tolerance. The resulting optimal values of the support pressure are $p_{min}^{*}=-0.5$ kPa (3.8 mmHg) and $p_{max}^{*}=6$ kPa (45 mmHg) for *fib* = 0.3 and $p_{min}^{*}=-0.6$ kPa (4.5 mmHg), $p_{max}^{*}=12$ kPa (90 mmHg) for *fib* = 0.5. For example, for the modeled case with 50% fibrosis in comparison with the control LV, the EF can be increased by 5% with only diastolic support, increased by 14% with only systolic support, and completely reach the value of the modeled control LV (65.6%) with the combination of the diastolic and systolic support. The end-diastolic pressure of 1.8 kPa (13.5 mmHg) cannot be decreased with only systolic support and can completely reach the value of the modeled control LV (7.5 mmHg) with only diastolic support as well as with the combination of the diastolic and systolic support. By increasing the value of the diastolic support, the end-diastolic hydrostatic stress becomes positive. This means that the myocardial tissue is extended and volumetric increase (possibly via a fluid inflow) is present.

## 4. Discussion

The simulation results show that, with an increased amount of fibrosis, the cardiac performance is reduced; specifically, a reduction in EF is accompanied by increases in ESV and EDP, as shown in Figures 3B,D,E. These results mirror the experimental studies in rats after myocardial infarction, where the infarct size was determined as the percentage of the fibrotic scar in the LV (21, 40). As depicted in Figure 3C, for an amount of fibrosis of 20%, EDV decreases by 11%. With a further increase in the amount of fibrosis, EDV remains nearly constant due to the combination of the stiffer myocardium and a higher EDP, as shown in Figure 3E.

It is worth noting that the alterations in EDP and EDV are caused solely by changes in the passive material properties, whereas the value of ESV is influenced by both the change in the stiffness as well as the reduced maximum active tension, i.e., contractility, which is in Equation (5) scaled by the amount of fibrosis. The absolute value of the hydrostatic stress depicted in Figure 3F increases nearly linearly with the amount of fibrosis. Its negative value represents a mechanical compression in the cardiac tissue modeled as a continuum. Theoretically, a high negative hydrostatic stress together with a high EDP might lead to compression and the closure of arterioles supplying the heart and then eventually to an under-perfusion. A more elaborate computational model accounting for the heart perfusion is needed to interpret the results quantitatively.

Considering the support pressure resulting from the difference between the ventricular pressure in a healthy and diseased LV, Figure 4C shows that the support pressure is sufficient for reaching the control EDV. We note that the maximum possible EDV was limited to that of the healthy rat. Therefore, even if the negative support pressure is higher than necessary, a normalization of the EDV is accompanied by a reduction in the EDP as depicted in Figures 4C,E. Due to the reduction in the EDP and enlargement of the LV, the hydrostatic stress becomes positive for all three fibrosis stages. This means that the tissue is under tension and better perfusion is expected. However, the observed decrease in ESV (Figure 4D) is marginal and only sufficient for the case *fib* = 0.1. Therefore, higher systolic support pressure is needed in order to reduce the ESV and eventually increase the EF, which is significantly below that of the control rat for higher amounts of fibrosis. For example, the resulting EFs are 61.1%, 55.2% for *fib* = 0.3, 0.5, respectively, compared to the control rat with EF = 65.6%.

**Figure 7:**
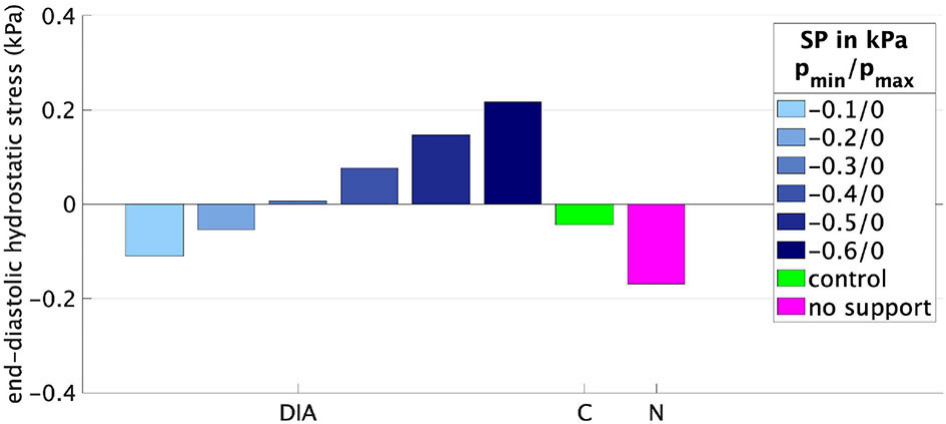
End-diastolic hydrostatic stress for different support pressures (SPs) applied to the rat LV with *fib* = 0.5. DIA, diastolic support; C, control rat LV; N, rat LV with *fib* = 0.5 without any support.

In the numerical test, that applied a constant minimum and maximum support pressure during the diastole and systole, respectively, the algorithm described in Section 2.2 is exemplarily performed for two fibrosis stages, namely *fib* = 0.3, 0.5. We started with supporting only the diastolic phase such that the negative value of the support pressure is increased until the desired EDV and EDP are reached up to a given tolerance. The EF can be increased by approximately 5% for both fibrosis stages, resulting in EF = 60.7%, 53.3% for *fib* = 0.3, 0.5, respectively. Even for the case with 50% fibrotic tissue, a relatively small support pressure of −0.6 kPa (−5.3 mmHg) is sufficient for regaining the desired EDV and EDP. Furthermore, as depicted in Figure 7, the compressive hydrostatic stress in the stiff fibrotic myocardium can be reduced and even changed into positive hydrostatic stress. We believe that this phenomenon could improve myocardial perfusion. However, as discussed above, this is currently only speculative and needs to be investigated. When only the systolic phase is supported, significantly higher positive support pressures are needed to normalize the systolic function, 6 kPa (45 mmHg) and 12 kPa (90 mmHg) for *fib* = 0.3, 0.5, respectively. Nevertheless, in this case, the desired EF of the control rat (65.6%) is not reached due to insufficient diastolic filling caused by the stiff fibrotic tissue. When using only the systolic support, a further increase in the support pressure would theoretically lead to the desired EF. However, the EDP would remain elevated, which has been identified as a potential predictor of heart failure (44–46). Whether solely the reduction in EDP would eventually lower the risk needs to be investigated further. The best option for normalizing EF and EDP turns out to be a combination of both, diastolic and systolic support. As demonstrated in Figures 5, 6, complete normalization of the functional parameters can be reached.

The present study aimed to explore the potential usefulness of diastolic support during the cardiac cycle of a rat LV suffering from restrictive cardiac disease. A simple computational model based on an investigation of mechanical support acting on the outer surface of the LV was chosen. However, there are some limitations and possibilities for future model development. First, the present study investigated the rat heart model and, to date, is not clinically applicable. However, as pointed out in the Introduction, some innovative approaches have investigated possible direct compression assist devices for clinical use in humans. Second, since it is difficult to develop a model that accounts for all influencing factors causing heart insufficiency, we initially chose a model in which we can change both the diastolic and the systolic function of a ventricle by changing one parameter, namely the amount of fibrosis. Besides this, various other factors must be considered in the future to more realistically mimic the remodeling process. These include the effects of geometric changes (in particular those due to ventricular dilatation or hypertrophy), the fact that most failing hearts are rather dilated than restrictive (especially in Paediatrics), the fact that myocardial infarction and ischaemic heart disease are only one among multiple causes of heart failure, the influence of arrhythmias and synchronization, the ever-changing metabolic needs, the fluid status of the patient impacting the preload of the heart, and much more. Third, for a better understanding of the interaction between the heart and a direct cardiac compression device, instead of the prescribed support pressure, a complete direct cardiac compression device should be modeled. Here, one possibility would be to use mechano-active materials, such as biocompatible dielectric elastomer actuators (47) that compress and expand when a voltage is applied. Their relatively large (more than 40%) expandability would be beneficial for generating the support pressure needed during the diastole (48). Fourth, in future development, electromechanical coupling and in particular electromechanical feedback would possibly play a role with respect to the interaction of the heart and the direct cardiac compression device.

## 5. Conclusion

A computational model for different amounts of fibrosis in the rat LV is presented. Based on this model, we investigate how a support pressure acting on the outer surface of the diseased LV influences the cardiac performance quantified via the EF, EDP, and ESV, as well as the hydrostatic stress in the cardiac tissue. We conclude that a negative support pressure during diastole combined with a positive support pressure during systole can normalize the modeled diastolic and systolic function of the rat LV at different fibrosis stages. Although not investigated in this study, it is tempting to assume that the negative diastolic support pressure could potentially improve cardiac perfusion. Furthermore, we adjusted the value of the support pressure so that functional parameters of the healthy rat LV are restored. We conclude that cardiac assist devices without direct blood contact and with a simultaneous diastolic and systolic support functionality present a potentially interesting therapy for heart failure in restrictive LV physiology, as that is resulting from myocardial scars after ischaemic insults.

## Data Availability Statement

The raw data supporting the conclusions of this article will be made available by the authors, without undue reservation.

## Author Contributions

DM mainly contributed to this study concerning the writing of the original manuscript, deriving the equations, modeling, simulation, and post-processing and visualization of the results. DH contributed to the fiber modeling and the revision of the manuscript. DB contributed to initial simulations and post-processing of simulation results. MW contributed to the acquisition of the publication fee support and advice for the medical part. SL and MA contributed to the conception and design of the study and the revision of the manuscript and provided the leadership responsibility for the technical and medical parts, respectively. MA contributed by writing and improving the medical parts of the manuscript. All authors contributed to the article and approved the submitted version.

## Funding

This study was funded by the Klaus Tschira Stiftung Grant 00.289.2016. The authors acknowledge financial support by Deutsche Forschungsgemeinschaft and Friedrich-Alexander-Universität Erlangen-Nürnberg within the funding programme “Open Access Publication Funding”.

## Conflict of Interest

The authors declare that the research was conducted in the absence of any commercial or financial relationships that could be construed as a potential conflict of interest.

## Publisher's Note

All claims expressed in this article are solely those of the authors and do not necessarily represent those of their affiliated organizations, or those of the publisher, the editors and the reviewers. Any product that may be evaluated in this article, or claim that may be made by its manufacturer, is not guaranteed or endorsed by the publisher.

## Figures and Tables

**Table A1:** Material parameters used in the numerical examples.

Active stress	*t*_*ed*_ = 60 ms, *t*_*ps*_ = 120 ms, $\alpha_{min}=-0.1{\text{ms}}^{-1}$, $\alpha_{max}=0.1{\text{ms}}^{-1}$, γ = 25 ms, *T*_*max*_=70 kPa,
Passive stress	*a* = 1.665 kPa, *b* = 1.237 (-), *a*_*f*_ = 7.822 kPa, *b*_*f*_ = 0.008 (-), *a*_*s*_ = 0.0 kPa,
	*b*_*s*_ = 0.0 (-), *a*_*fs*_ = 1.342 kPa, *b*_*fs*_ = 9.178 (-)
Windkessel model	*R*_*p*_ = 15 kPa μL^−1^ms, *R*_*v*1_=0.1 kPa μL ^−1^ms, *R*_*v*2_=0.067 kPa μL^−1^ms,
	*C* = 40 μL kPa ^−1^ ms^−1^, *t*_*a*_ = 40 ms
Support pressure	*t*_*es*_ = 150 ms, *t*_Δ_ = 10 ms, *tol*_1_ = 1μL, *tol*_2_ = 0.1 kPa

## Appendix

### Active Tension

Based on the study by Pfaller et al. (38), the evolution of the active tension *T* reads as

$$\begin{array}{l} Ṫ\left(t\right)=-|a\left(t\right)|T\left(t\right)+T_{max}|a\left(t\right){|}_{+} \end{array}$$

with activation function *a*, the maximum value of the active stress *T*, and the function |*a*(*t*)|_+_ = max(*a*(*t*);0). The activation function *a*(*t*) is modeled by

$$\begin{array}{l} a\left(t\right)=\alpha_{max}\cdot f\left(t\right)+\alpha_{min}\cdot \left(1-f\left(t\right)\right) \end{array}$$

with maximum and minimum activation rates α_*max*_ and α_*min*_, respectively, and functions

$$\begin{array}{ll} f\left(t\right) & =S^{+}\left(t-t_{ed}\right)\Delta S^{-}\left(t-t_{ps}\right),\\ S^{\pm }\left(\Delta t\right) & =\frac{1}{2}\left(1\pm \tanh\left(\frac{\Delta t}{\gamma }\right)\right) \end{array}$$

with steepness γ and descending and ascending sigmoid functions *S*^+^ and *S*^−^, respectively. The indicator function *f*∈(0, 1) indicates systole. The times *t*_*ed*_ and *t*_*ps*_ model the end-diastolic and the peak-systolic times, respectively.
